# An Analysis and Simulation of Security Risks in Radar Networks from the Perspective of Cybersecurity

**DOI:** 10.3390/s25175239

**Published:** 2025-08-23

**Authors:** Runyang Chen, Yi Zhang, Xiuhe Li, Jinhe Ran

**Affiliations:** 1 College of Electronic Engineering, National University of Defense Technology, Hefei 230037, China; chenrunyang19@nudt.edu.cn (R.C.); zy_zhangyi@nudt.edu.cn (Y.Z.); ranjinhe17@nudt.edu.cn (J.R.); 2 Anhui Province Key Laboratory of Electronic Environment Intelligent Perception and Control, Hefei 230037, China

**Keywords:** radar networks, risk analysis, MITM, FDIA

## Abstract

Radar networks, composed of multiple radar stations and a fusion center interconnected via communication technologies, are widely used in civil aviation and maritime operations. Ensuring the security of radar networks is crucial. While their strong anti-jamming capabilities make traditional electronic countermeasures less effective, the openness and vulnerability of their network architecture expose them to cybersecurity risks. Current research on radar network security risk analysis from a cybersecurity perspective remains insufficient, necessitating further study to provide theoretical support for defense strategies. Taking centralized radar networks as an example, this paper first analyzes their architecture and potential cybersecurity risks, identifying a threat where attackers could potentially execute false data injection attacks (FDIAs) against the fusion center via man-in-the-middle attacks (MITMAs). A threat model is then established, outlining possible attack procedures and methods, along with defensive recommendations and evaluation metrics. Furthermore, for scenarios involving single-link control without traffic increase, the impact of different false data construction methods is examined. Simulation experiments validate the findings, showing that the average position offset increases from 8.38 m to 78.35 m after false data injection. This result confirms significant security risks under such threats, providing a reference for future countermeasure research.

## 1. Introduction

A radar network is formed by strategically deploying multiple radars and connecting them through network communication technology. It employs a fusion center (network central station) to exercise unified control and management over the radar sites and utilizes data fusion algorithms to process and integrate data from each site. This setup enables the full exploitation of the advantages of radar clusters and information fusion, playing a crucial role in many fields [1]. In a radar network, network communication technology greatly facilitates information exchange between individual radar sites and the central station. However, it also significantly enhances the openness of the entire radar network system, thereby increasing its security risks accordingly. For instance, on 5 and 10 June 2014, multiple aircraft disappeared from the ATC radar system in Europe, each time for up to 25 min. It was believed that the ATC radar system might have been subjected to a hacker attack [2]. In a broader sense, if a radar network is regarded as a type of Cyber–Physical System (CPS), it also faces the same cybersecurity threats as a CPS. For example, in 2010, the “Stuxnet” virus attacked Iran’s nuclear facilities [3]. In 2015, the Ukrainian power grid suffered a cyber attack, resulting in widespread power outage [4]. All these cases clearly demonstrate that radar networks may be susceptible to cyber attacks, the consequences of which are unacceptable.

Compared to traditional electronic countermeasures, cyber attacks are lower-cost, more stealthy, and offer precise, controllable effects with potentially severe consequences. To mitigate such risks, it is essential to conduct cybersecurity-focused risk analysis for radar networks. However, research in this field remains in its early stages. The network security inspections conducted by Boris Svilicic et al. on shipborne radars on two vessels [5], as well as Eduardo Esteban Casanovas’s analysis of the ASTERIX radar protocol revealing vulnerabilities [6], both indicate the existence of cybersecurity risks in radar systems. Konrad Wolsing et al. [7] classified potential cyberattacks on maritime radars and constructed a simulation environment and datasets. Following on from this, multiple researchers [8,9] have conducted intrusion detection research. However, these studies focus on local area networks formed by the connection of internal equipment on ships, rather than radar networks composed of different radars [10,11]. The network security problem of a radar network composed of multiple radars has not been given enough attention. As a key infrastructure, it should be studied more. Therefore, the research object of this paper is a radar network composed of multiple radars. Walmor Cristino Leite Junior et al. [12] proposed that it is feasible for an attacker to use a radar system as a breach point to send commands to a ship in order to activate the malicious code previously installed on the vessel. Shai Cohen et al. [13] provided a comprehensive list of potential risks faced by typical radar systems, including risks in the fields of electronic warfare and cybersecurity, and identified attack scenarios related to typical radar systems. Nevertheless, the research is limited to individual radars and lacks a holistic study of “radar networks,” failing to consider the impacts of data fusion algorithms, tracking filtering algorithms, and track initiation algorithms at the fusion center. The main difference between a radar network and a single radar is its “network” attribute. The “network” attribute enables the radar network to obtain information from multiple radar stations and then fuse it to generate intelligence with higher reliability. This involves the above algorithms, which makes the radar network greatly different from a single radar. Therefore, it is not enough to rely only on the previous network security research for a single radar; there must be targeted research on the “radar network” and the impact of these algorithms. Studies [14,15,16] also have such limitations. It is necessary to extend relevant theories from individual radars to radar networks. Nova Hadi Lestriandoko et al. [17] considered the issue of radar networks and proposed a chaotic algorithm to generate pseudo-random numbers for securing internet communication between radar stations and the host station. However, this study lacks in-depth research on attack methods and implementation means. Chaoqun Yang et al. [2] considered the impact of false data injection attacks on radar networks on fusion results but fell short of analyzing cyberattack threats comprehensively. There is also a lack of analysis on possible attack methods, means, potential construction methods, and the potential harm they could cause. This has led to a lack of an intuitive and in-depth understanding of the network security threats faced by radar networks, and it is not clear from which channels and in what ways malicious attackers may launch attacks that are passive for the defender. As the saying goes, “One cannot defend without knowing how to attack.” The detailed modeling of attacks is a prerequisite for attack–defense confrontation. Only by accurately modeling attack capabilities can effective defenses be constructed.

The main contributions of this paper are as follows:

1. Complementing Threat Modeling in Radar Network Security. This study supplements research on threat modeling in radar network cybersecurity, revealing the security risks of false data injection attacks (FDIAs) against fusion centers via man-in-the-middle attacks (MITMAs). It provides a detailed analysis of potential attack procedures and implementation methods, along with defensive recommendations, laying a theoretical foundation for future defense research.

2. Exposing Security Threats in Specific Attack Scenarios. This paper demonstrates that attackers can achieve effective attacks by merely tampering with single-link control without increasing network traffic, highlighting the feasibility of FDIAs in such scenarios. It points out the limitations of traditional traffic monitoring-based defense mechanisms in countering such threats, providing a reference for developing more efficient and precise defense solutions.

3. Quantitative Evaluation and Simulation of Attack Impact. This study proposes evaluation metrics for attack effectiveness and verifies the disruptive impact of false data injection through simulations. The experimental results show a significant increase in average position offset after injection, confirming the vulnerability of radar networks to such cybersecurity threats.

## 2. Cybersecurity Threats Faced by Radar Networks

**Figure 1:**
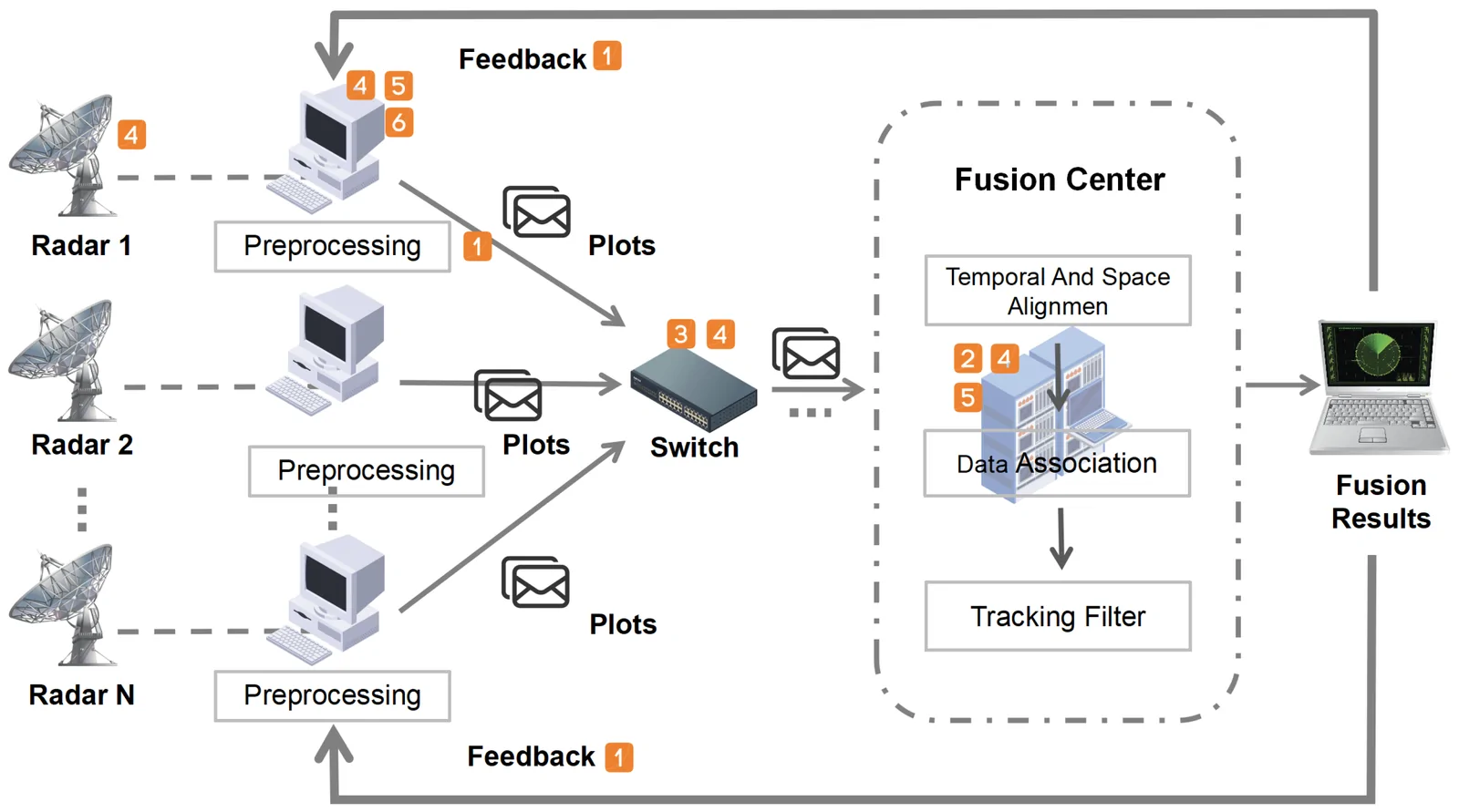
A schematic diagram of the centralized radar network. The orange boxes in the diagram indicate potential cyber attacks that the locations may be subjected to.

A schematic diagram of the centralized radar network is shown in Figure 1. The entire network consists of multiple radar sites, a fusion center, and communication infrastructure. Each individual radar operates independently, conducting only detection and preprocessing tasks. The preprocessed raw plot data is then uploaded in its entirety to the fusion center, where centralized processes such as spatio-temporal alignment, data association, and tracking filtering are performed to generate a unified track. Typically, the fusion center provides real-time feedback after filtering to guide the radar sites in adjusting their detection areas. Centralized networks are usually deployed among radars of the same type or comparable accuracy, with a limited number of nodes.

Cyber attacks targeting radar networks include but are not limited to the below:

(1) **Man-in-the-Middle Attack (MITM)**: By hijacking the communication link between radar stations and the fusion center, the attacker intercepts and collects intelligence or modifies transmitted data before forwarding it, thereby achieving the goals of intelligence gathering or data manipulation [18,19,20].

(2) **False Data Injection Attack (FDIA)**: By injecting false plots or tracks into the fusion center, the attacker alters existing tracks or generates fictitious ones, affecting the fusion center’s ability to accurately locate targets or causing operator misjudgment [21,22,23].

(3) **Denial-of-Service Attack (DDoS)**: The communication channel between radar stations and the fusion center is flooded with excessive traffic to paralyze normal operations [24].

(4) **Supply Chain Attack**: Backdoors or malicious firmware are embedded into radar systems, communication equipment, or fusion center servers during the supply chain process.

(5) **Firmware/Software Exploitation**: Unpatched vulnerabilities (e.g., buffer overflow, default credentials) are exploited in radar stations or fusion center servers to gain control.

(6) **Malicious Code Implantation**: Trojans are inserted via maintenance interfaces or remote update channels to manipulate radar parameters or disable functionalities.

The number in Figure 1 represents the position where the above attack methods may occur. The numbers 1–6 represent the above 6 attacks respectively.

An MITM attack requires the attacker to infiltrate the target communication link and parse the communication protocol, but it offers controllable and covert operational effects, making it an effective means to achieve false data injection attacks. A DDoS attack disrupts normal communication through traffic flooding, but its high traffic volatility makes it easily detectable and less covert, leaving it vulnerable to countermeasures. The supply chain attack necessitates pre-installing backdoors and malicious firmware in supply chain devices, involving a wide scope and posing significant implementation challenges. Exploiting firmware or software vulnerabilities requires knowledge of the target system’s operating model and, in some cases, zero-day vulnerabilities, resulting in high costs and difficulty. Malicious code implantation via maintenance interfaces or remote update channels is relatively feasible.

Among these, MITM and FDIA strike the optimal balance of feasibility, stealth, and impact, making them primary threats. Therefore, this paper specifically focuses on these attack methods, modeling the implementation of false data injection attacks via man-in-the-middle attacks.

## 3. Threat Models and Defense Measures

**Figure 2:**
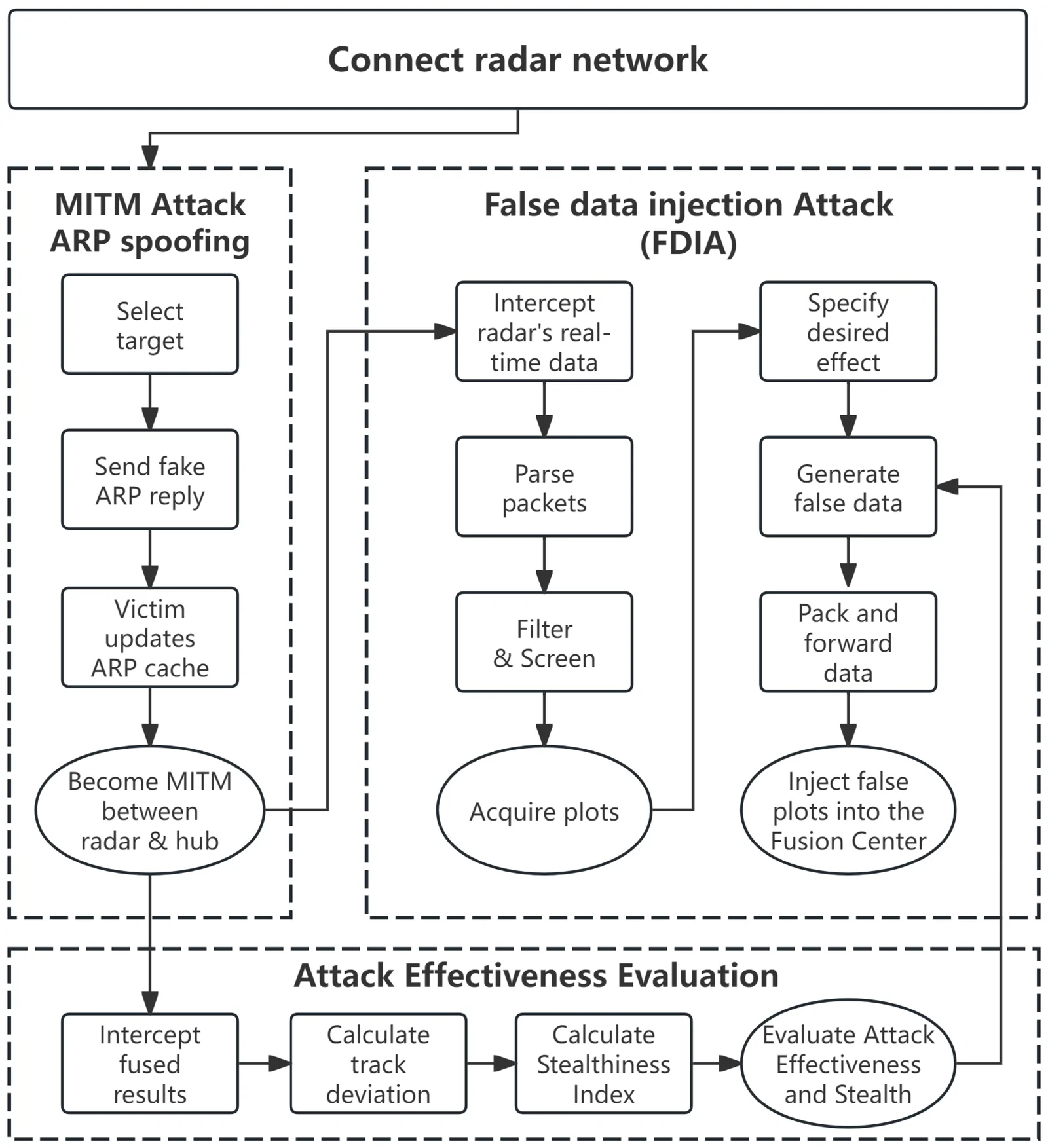
The threat model for conducting man-in-the-middle attacks and false data injection attacks on radar networks. (The rectangle represents an action and ellipse represents an achievement).

Figure 2 shows the threat model for conducting man-in-the-middle attacks and false data injection attacks on radar networks. Overall, the entire implementation process comprises four major segments: connecting to the radar network’s communication channel, executing the man-in-the-middle attack, launching the false data injection attack, and evaluating the attack’s effectiveness. We will now delve into a detailed analysis of the implementation mechanisms for each step and provide recommendations for defensive measures.

### 3.1. Connect Radar Network

Network communication between radar networks can be categorized into wired and wireless types based on the transmission medium. Fixed radar sites typically use high-bandwidth fiber-optic links due to long distances between stations. However, for mobile ad-hoc radar networks, wireless transmission via electromagnetic radiation is generally used. Each transmission method presents distinct challenges.

If a wired network such as a fiber-optic network is breached, there are two potential scenarios:

Direct Connection: Attackers may directly connect to the main switch of the fusion center or to the radar components at individual radar stations. For instance, a malicious device could be connected through maintenance interfaces or user terminals to the ports of the main switch or the debugging interfaces of radar components. Radar data transmission networks often have external interfaces reserved for maintenance and troubleshooting, making such intrusions feasible. Additionally, attackers may employ infected USB drives or other mobile storage devices to indirectly infiltrate the network through a “ferrying” technique.

Interception Between Radar Station and Fusion Center: Attackers can position their devices between the radar station and the main switch of the fusion center. They can physically tap into the fiber-optic cable by stripping its outer sheath and coating or by using hydrofluoric acid to etch away part of the fiber’s cladding [25]. Then, using optical coupling technology, they can pick up leaked optical signals with high-sensitivity optical detection equipment, amplify them, convert them into electrical signals, and, finally, decode them to eavesdrop on the information carried by the optical signals. When sending information, they can inject high-power, modulated optical signals of the same wavelength into the fiber to achieve transmission.

Infiltrating a wireless network requires proximity to the signal coverage area, protocol knowledge, and encryption cracking capability. Since wired networks often assume that they are physically isolated (air-gapped), they frequently transmit data in plaintext, making them more vulnerable to security breaches [26].

Defense Recommendations: First, physical security must be strengthened by strictly controlling access to critical equipment such as the main switch and radar component debugging interfaces. Access control systems, video surveillance, and log auditing should be implemented to ensure that only authorized personnel can approach these devices. Second, port security measures must be enabled, such as MAC address binding on switch ports, to prevent unauthorized device connections. Finally, transmitted data must be encrypted and digital signature technology must be incorporated to prevent tampering.

### 3.2. Man-in-the-Middle Attack via ARP Spoofing

**Figure 3:**
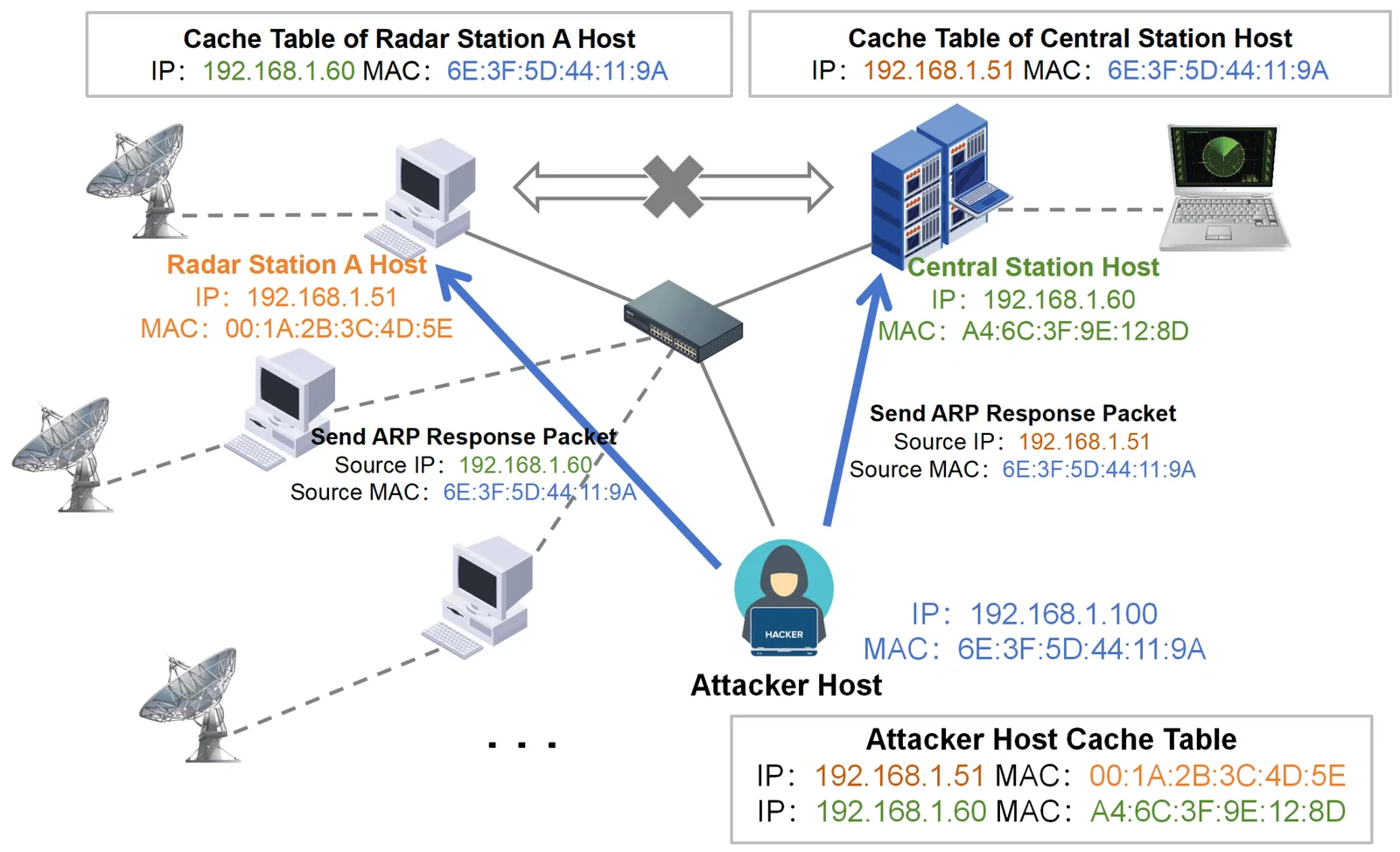
Schematic diagram of implementing man in the middle attack using ARP spoofing.

This involves impersonating an intermediary between the radar station server and the fusion center server. Attackers typically employ techniques such as ARP spoofing, DNS (Domain Name Server) spoofing, and SSL (Security Socket Layer) stripping. In the context of radar networks, where communication between radar stations and the fusion center’s servers usually relies on IP addresses, ARP spoofing presents a higher success rate. Therefore, this section focuses specifically on the method of achieving a man-in-the-middle attack through ARP spoofing. Figure 3 shows an example of a man-in-the-middle attack using ARP Spoofing.

In an ARP spoofing attack, the attacker sends falsified ARP (Address Resolution Protocol) messages over a local area network [27]. These messages associate the attacker’s MAC address with the IP address of a legitimate network entity, such as the fusion center’s server. As a result, traffic intended for the legitimate server is redirected to the attacker’s machine. The attacker can then intercept, modify, or even block the data being transmitted between the radar station and the fusion center, effectively acting as a man-in-the-middle. This enables the attacker to gain unauthorized access to sensitive information, disrupt communication, or inject malicious data into the radar network [28,29].

Specific actions include the below:

(1) Select Attack Targets: The attacker first selects a radar station host (Host A) within the radar network and the fusion center host (Host B) as the targets for the attack.

(2) Forging ARP Responses: The attacker sends forged ARP (Address Resolution Protocol) packets to the target devices (Host A and Host B). To Host A, the attacker’s packet claims: “The MAC address of Host B is the attacker’s MAC (impersonating Host B).” To Host B, the packet claims: “The MAC address of Host A is the attacker’s MAC (impersonating Host A).”

An example of a forged ARP packet sent by the attacker:

Sender MAC: Attacker’s MAC

Sender IP: Host B’s IP (to deceive Host A)

Target MAC: Host A’s MAC

Target IP: Host A’s IP

(3) Updating ARP Cache: Upon receiving the forged ARP responses, the target devices (Host A and Host B) update their ARP cache tables, binding the attacker’s MAC address to the legitimate IP addresses. As a result, data intended from Host A to Host B is actually sent to the attacker’s MAC address and data intended from Host B back to Host A is also sent to the attacker’s MAC address.

(4) Becoming the Man-in-the-Middle and Achieving Traffic Hijacking: By successfully manipulating the ARP caches, the attacker positions themselves as the intermediary between Host A and Host B, enabling them to intercept, modify, or block the data traffic between the two hosts.

The feasibility of ARP spoofing is quite high, especially in local area networks (LANs) lacking adequate protective measures. One contributing factor is that radar networks often assume that they are physically isolated, leading to a lack of corresponding security measures in their network design. Consequently, once an attacker gains access to the LAN, the likelihood of a successful ARP spoofing attack is significantly increased.

Defense Recommendations: Implement static ARP binding on critical devices such as gateways and servers by manually configuring IP-MAC mappings to prevent forged ARP responses. Alternatively, deploy ARP monitoring tools to detect and alert on anomalous ARP requests and responses in real time.

### 3.3. False Data Injection

**Figure 4:**
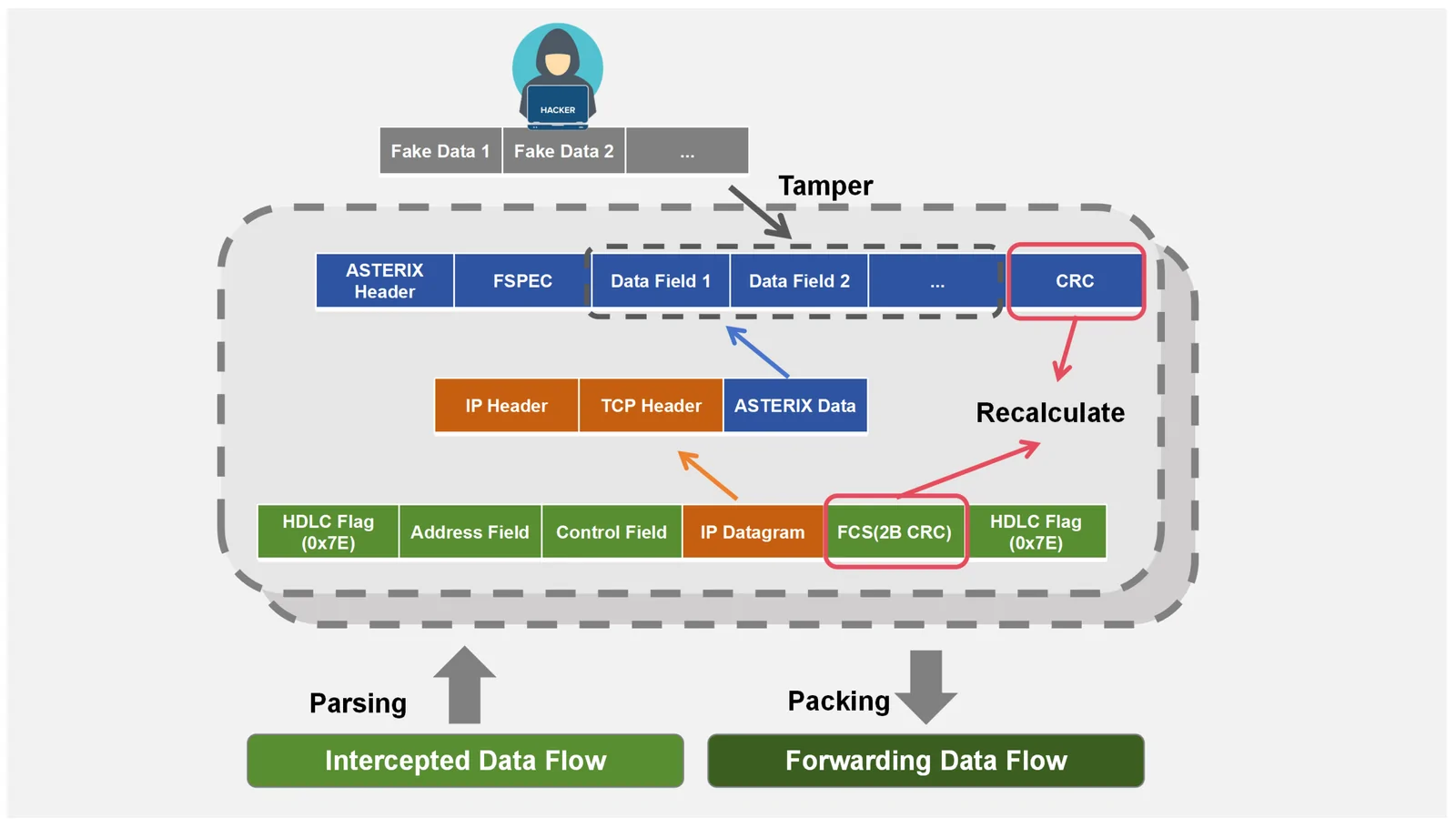
Schematic diagram of false data injection process.

False data injection includes the following three parts, and Figure 4 is a schematic diagram of this process.

(1) Interception of Real-time Data: The attacker will intercept the data packets uploaded from the host at the radar site to the server at the fusion center site, as well as the fused tracks, and send information dispatched from the fusion center server to the radar site.

(2) Parsing, Filtering, and Screening: Taking the civil aviation radar network as a reference, its data transmission network is an SDH network. The data link layer utilizes data packets specified by the HDLC protocol, with the information field containing radar data encapsulated using the ASTERIX protocol [30]. The network layer employs IP addresses and the transport layer adopts the TCP protocol. Therefore, the attacker will parse the data packets according to these protocols, filter out irrelevant fields such as flag fields, screen out the information fields, and extract the plot information detected by the radar site.

(3) False Data Construction: Attackers may use intercepted data as a blueprint to construct false data. Since the target data is encapsulated with headers from different protocol layers during transmission, they may retain these headers and only tamper with the plot information in the ASTERIX protocol and the CRC checksum. By reverse-engineering the protocol, they can identify and modify the critical fields representing plot information. This approach minimizes effort, avoids formatting errors, and maximizes attack success rates, representing a primary threat. The tampering process is illustrated in Figure 4.

Defense recommendation: Multi-source data consistency checks are implemented, such as redundant sensor validation, by comparing data from multiple radar stations. Data points deviating from the majority are flagged as anomalies. Even if spoofed data is injected, its impact will be diluted among multiple sources. Alternatively, physical model verification can be used to detect data that violates kinematic laws.

### 3.4. Attack Effect Evaluation

To ensure the effectiveness of an attack, the attacker may evaluate the attack results in order to adjust the magnitude of the offset or the method of tampering.

Existing research has not yet specifically addressed the evaluation of attack effectiveness for cyber attacks against radar networks. Current studies primarily focus either on evaluating jamming effectiveness in electronic countermeasures (ECMs) against radar systems or on assessing the stealthiness of cyber attacks in internet-based scenarios. However, neither approach provides a comprehensive evaluation framework for cyber attacks on radar networks.

The ECM-oriented approach, influenced by traditional electronic warfare concepts, emphasizes attack “effectiveness” (disruption capability) while overlooking “stealthiness”—a crucial characteristic for cyber attacks. Conversely, internet-based research considers “stealthiness” but fails to account for the attack’s actual impact on radar performance, the essential “effectiveness” metric.

Building upon these existing works, we propose that evaluating cyber attacks against radar networks requires the simultaneous consideration of both effectiveness and stealthiness. Accordingly, we developed quantitative metrics for both attack effectiveness and stealthiness to establish a reference framework for future research in this domain.

Effectiveness refers to whether an attack has achieved its intended goal. The measurement of effectiveness is related to the purpose of the attack. For creating false tracks, it is necessary to determine whether the number of generated false tracks meets the requirements. For altering existing tracks, the primary indicator is the offset, that is, the magnitude of the deviation between the attacked track and the actual track.

If the attack goal is to change the real track, the track offset is selected as the indicator. The accumulated position offset between the fused track and the real track is calculated as the method for computing the track offset.

Assume that the track before tampering is *U* and the plot at time *i* is $P_{i}$; then,

(1) $$U=\{P_{1},P_{2},\ldots ,P_{i},\ldots ,P_{N}\}$$

(2) $$P_{i}=\left(a_{i},b_{i}\right)$$

where $a_{i},b_{i}$ represent the horizontal and vertical coordinates of the target plot at time *i*. Similarly, the tampered track is *D* and the plot at time *i* is $Q_{i}$:

(3) $$D=\{Q_{1},Q_{2},\ldots ,Q_{i},\ldots ,Q_{N}\}$$

(4) $$Q_{i}=\left(c_{i},d_{i}\right)$$

where $c_{i},d_{i}$ represent the horizontal and vertical coordinates of the target plot at time *i*.

The specific formula for accumulated position offset (ACPO) is

(5) $$ACPO=\sum_{i=1}^{N}\sqrt{{\left(a_{i}-c_{i}\right)}^{2}+{\left(b_{i}-d_{i}\right)}^{2}}$$

By taking the average of the accumulated position offset, we can obtain the average position offset (AVPO):

(6) $$AVPO=\frac{1}{N}\sum_{i=1}^{N}\sqrt{{\left(a_{i}-c_{i}\right)}^{2}+{\left(b_{i}-d_{i}\right)}^{2}}$$

The track offset can be measured using the accumulated position deviation standard. In the specific calculation process, if the attacker cannot obtain the real position of the target, the plot information uploaded by the intercepted radar station will be used as the real track of the target. It is calculated after matching according to the real track batch numbers in the data and the timestamps within the plots.

If the attack goal is to create false tracks, then the false-track generation success rate is selected as the indicator. This indicator measures the ability of the attack command to successfully trigger the radar network to generate initial false tracks.

(7) $$P=\frac{N_{detected}}{N_{attack}}$$

where $N_{detected}$ is the number of false tracks that the attacker attempts to inject (attack plan). $N_{attack}$ is the number of false tracks detected and tracked by the radar network system.

Stealthiness refers to how easily the attack can be detected. Specific indicators include the number of packets that need to be intercepted and forwarded to achieve the attack effect, the amount of traffic variation, and the delay time. Two indicators are now proposed to measure the attack effectiveness.

Set the stealthiness index *S* as the measurement indicator.

(8) $$S=\alpha \cdot e^{-\delta N_{min}}+\beta \cdot e^{-\theta \eta }+\gamma \cdot e^{-\mu T_{delay}}$$

The closer *S* is to 1, the more stealthy the attack is; the closer *S* is to 0, the more likely the attack is to be exposed.

$N_{min}$ is the packet operation volume (POV), defined as the minimum number of data packets that the attacker needs to intercept, tamper with, and forward. It is affected by the complexity of the radar data fusion algorithm, the accuracy requirements of the attack target, and the radar network topology.

η is the traffic variation rate (TVR), defined as the ratio of the additional traffic caused by the attack to the baseline traffic. The calculation formula is

(9) $$\eta =\frac{\lambda_{attack}-\lambda_{normal}}{\lambda_{normal}}\times 100\%$$

where $\lambda_{normal}$ is the normal traffic and $\lambda_{attack}$ is the total traffic during the attack.

$T_{delay}$ is the attack latency, defined as the total time from intercepting data packets to completing the injection.

(10) $$T_{delay}=T_{intercept}+T_{tamper}+T_{transmit}$$

where $T_{intercept}$ is the interception delay, $T_{tamper}$ is the tampering delay, and $T_{transmit}$ is the transmission delay.

Where α, β, and γ are weighting coefficients, which reflect the importance attached to various influencing factors. The allocation of weights is based on a prior assessment of the exposure risk associated with attack behaviors. Packet tampering (POV) exhibits the highest immediacy and is therefore assigned the greatest weight. Traffic variation (TVR) and delay ($T_{delay}$) possess relatively stronger concealment characteristics, resulting in progressively decreasing weights. Values for α, β, and γ can be temporarily set to 0.5, 0.3, and 0.2, respectively.

δ, θ, and μ are decay factors. Decay factors are applied to eliminate the influence of differences in scale among the metrics. Factors δ and θ can be temporarily set to 1.0, while μ can be temporarily set to 1000. This simplified approach is a prudent measure taken due to current data limitations and can be further calibrated through experimentation in the future.

### 3.5. Realistic Feasibility Analysis

The Stuxnet attack on industrial control systems and the cyberattack on Ukraine’s power grid demonstrate the significant threats that cyberattacks pose to critical infrastructure. These attacks are highly targeted and designed to cause severe real-world consequences, proving that environments not connected to the internet, along with existing cyber defenses, cannot guarantee protection against malicious actors.

Radar networks, such as those for civil aviation, constitute critical infrastructure directly linked to flight safety. Typically, these radar networks are also not connected to the internet. However, the precedents set by the aforementioned incidents serve as a stark reminder of the potential risks. This is especially concerning given that some radar networks possess functionalities for remote access and control. Should terrorist organizations, hacktivists, or cybercriminals launch a coordinated attack, it could result in catastrophic security incidents.

These malicious actors could emulate the Stuxnet attack, using removable flash drives as infection vectors. They could then exploit interfaces intended for remote control, maintenance, or debugging to covertly infiltrate the target network or device, gain control of the equipment, and then carry out attacks; this is highly likely to happen in reality.

## 4. Classification and Hazards of False Data Construction Methods

### 4.1. Single Link Control Scenario

In practical attacks, it is impossible for an attacker to hijack all links within a local area network. This is because ARP spoofing requires the attacker to continuously send forged ARP response packets to maintain the deceptive state. If an attacker attempts to hijack all links, they would need to simultaneously spoof the ARP tables of a large number of hosts, which could lead to network congestion, abnormal traffic patterns, and a significantly increased risk of exposure. Moreover, hijacking all links places enormous demands on the attacker’s device performance as they would need to handle bidirectional traffic (forwarding or tampering) on all hijacked links. This imposes extremely high requirements on the attacker’s computational and bandwidth resources, making it particularly challenging to achieve in high-speed networks, such as those used for radar data transmission. Therefore, in practice, attackers typically choose to control only a single, specific link.

Consequently, this paper focuses on a scenario where an attacker has already hijacked the communication link between one radar station and the fusion center. Due to the lack of integrity protection, the attacker is capable of reading and modifying radar communications. Moreover, the attacker conducts modifications solely based on intercepted data, carrying out attacks under stealthy conditions without increasing network traffic. This paper enumerates several possible data tampering methods and analyzes their potential hazards.

(1) Null Value (Data Erasure): This involves intercepting and dropping data packets. The effect is to block the radar station from uploading target plots to the fusion center. From the fusion center’s perspective, it appears that the radar station did not detect any targets at that moment. Consequently, the radar network system receives less data, affecting its ability to utilize data redundancy for precise target localization and reducing the radar network’s detection effectiveness to some extent. In this scenario, targets located in the overlapping areas of radar surveillance are likely to still be detected normally, while targets detected only by the affected radar station will not be detected by the radar network. From the fusion center’s viewpoint, it is difficult to detect the attack on the radar display if the traffic transmitted by each station is not checked or if the detection results of the affected radar station are not displayed separately. However, this attack method has limited effectiveness. The radar network can still detect the majority of tracks, and the attacker cannot ensure whether the flight target they intend to conceal is only tracked by the current radar station. Other radar stations may still have detected the target.

(2) Offset: This involves adding an offset to each coordinate, causing the entire plot to shift. By adding an offset to the position information of the plots uploaded by the radar station, the entire track is shifted. If the offset is sufficiently large, the fusion center will perceive it as a new track and initiate track processing, creating a “shadow track”. This method of generating additional tracks does not produce significant abnormalities in traffic. For the attacker, tracks constructed from false data that are translated based on the original plot information that conforms to the motion characteristics of flight targets. The speed, direction, and overall flight path are logical and not easily filtered out by the fusion center. This approach can also create the illusion of multiple targets in the same batch.

If the offset is small, the fusion center will regard the tampered plot (false point) as belonging to the same target, and, generally, only one track will be displayed. In this case, the false plot and the real plot will be jointly input into the tracking and filtering algorithm, causing deviations in the fusion results. If the offset is designed reasonably and increased progressively, it can make the fused and estimated plots deviate. This will lead to a significant divergence between the predicted track of the flight target and its actual track, until the real plot information from other radar stations falls outside the association gate.

(3) Scaling: Scaling involves multiplying each coordinate component of the target’s position data by a constant factor. This manipulation results in a new, false track that is parallel to the original real track but scaled proportionally. The spacing between plots is also scaled by the same factor. Since the radar scanning cycle and the time intervals between radar plots remain unchanged, the change in distance directly affects the perceived speed of the target. Through scaling, an attacker can generate a new track and alter the apparent speed of the target. If the scaling ratio is small, the generated false track will be close to the original track, causing the plots to be associated with the same target by the fusion center. This can lead to a continuous, erratic pattern in the fused track, compromising the precision of target localization.

(4) Flipping: Flipping involves taking the negative of each coordinate component of the target’s position data, effectively mirroring the track across the origin (in this case, the radar station). At the fusion center, this manipulation generates a false track that is symmetrical to the original track with respect to the radar station. The effect is similar to that of a large-offset translation as it creates a distinct, albeit mirrored, track that can mislead tracking algorithms.

The four simple tampering methods mentioned above can create false tracks or cause slight deviations in existing tracks. Research also found that using advanced comprehensive strategies, such as periodically injecting false data, linearly increasing deviations over time, and periodically reversing deviation directions, could significantly degrade a radar network’s tracking performance, posing substantial security risks.

To illustrate this risk, we provide a simple example as a research template:

1. **Set the goal**: The attacker aims to pollute the state estimation of target *M*’s track by associating false plots with the real target, causing position deviation or track jitter.
2. **Select the tampering method**: The attacker chooses offset as the data tampering method.
3. **Design the attack method**: The attacker adopts a method of *p* times of inactivity and *q* times of attack. Inject offsets periodically to avoid track splitting due to long-term offsets. Let the start time of the *i*-th action cycle be $t_{i}$; then, we have
  (11) $$t_{no\_attack}=\left\{t_{i},t_{i}+T,t_{i}+2T,\ldots ,t_{i}+\left(p-1\right)T\right\}$$
  (12) $$t_{attack}=\left\{t_{i}+pT,t_{i}+\left(p+1\right)T,t_{i}+\left(p+2\right)T,\ldots ,t_{i}+\left(p+q-1\right)T\right\}$$
  where $t_{no\_attack}$ is the set of inactivity moments, $t_{attack}$ is the set of attack moments, and *T* is the interval time, which is determined by the data upload period of the radar station.
  If the current moment $t\in t_{no\_attack}$, let ΔP=0 and jump to step 8. If $t\in t_{attack}$, then continue to execute this process.
4. **Obtain the target state**: The attacker intercepts the state measurement value $P_{t}=\left(x_{t},y_{t}\right)$ of the target *M* uploaded by the radar station at the current time *t* and combines with the intercepted $P_{t-T}=\left(x_{t-T},y_{t-T}\right)$ at time t−T to predict the velocity vector V of the target *M*’s track at the current time *t*. Assuming that the target *M* moves in a uniform linear motion, we have
  (13) $$V=(x_{t}-x_{t-T},y_{t}-y_{t-T})$$
5. **Determine the direction**: The attacker selects the offset direction d (unit vector) according to the tactical intention. If manufacturing lateral error, the direction perpendicular to the predicted velocity V is selected. At this time, we have
  (14) $$d=V_{⊥}=\left(-\frac{y_{t-T}-y_{t}}{\sqrt{{\left(x_{t}-x_{t-T}\right)}^{2}+{\left(y_{t}-y_{t-T}\right)}^{2}}},\frac{y_{t-T}-x_{t}}{\sqrt{{\left(x_{t}-x_{t-T}\right)}^{2}+{\left(y_{t}-y_{t-T}\right)}^{2}}}\right)$$
6. **Generate the offset amount ΔP**: Is is assumed that the radar network uses a correlation threshold *G* to determine whether two plots belong to the same target. Then, the basic offset amount is set as
  (15) $$\Delta P={\left(-1\right)}^{i}\cdot \delta \left(t\right)\cdot d\cdot G$$
  where ${\left(-1\right)}^{i}$ is used to periodically change the offset direction. δ(t) is used to control the offset ratio and is a time-varying function; specifically,
  (16) $$\delta \left(t\right)=\left\{\begin{array}{cc} 0, & t\in t_{no\_attack}\\ \alpha +k\cdot \left[\frac{t-(t_{0}+pT)}{T}\right], & t\in t_{attack} \end{array}\right.$$
  where α is the initial coefficient and *k* is the modulation slope. Threshold *G* can be obtained from the following equation:
  (17) $$P\left(s\leq G\right)={\;\int \int \;}_{x^{2}+y^{2}\leq G^{2}}\frac{1}{2\pi |S_{k}{|}^{1/2}}\exp\left(-\frac{1}{2}\gamma^{⊤}S_{k}^{-1}\gamma \right)dx\;dy=1-\beta$$
  where *s* represents the Euclidean distance between the predicted position and the measured position, $S_{k}$ represents the residual covariance matrix in the Kalman filter, and β represents the false alarm probability of the correlation process. If $S_{k}$ is a diagonal matrix and isotropic, the above formula can be simplified as
  (18) $$P\left(s\leq G\right)=\frac{1}{2\pi \sigma^{2}}\cdot 2\pi \cdot \sigma^{2}\left[1-\exp\left(-\frac{G^{2}}{2\sigma^{2}}\right)\right]=1-\exp\left(-\frac{G^{2}}{2\sigma^{2}}\right)=1-\beta$$
  then
  (19) $$G=\sigma \sqrt{-2\ln\beta }$$
7. **Increase stealthiness**: The attacker adds a random noise $n∼N(0,\delta^{2})$ that conforms to the radar accuracy model on ΔP; then, we have
  (20) ΔP=δ(t)·G·d+n
  At this time,
  (21) $$\Delta P=\left(\frac{\delta \left(t\right)\cdot d\cdot G\cdot \left(y_{t-1}-y_{t}\right)}{\sqrt{{\left(x_{t}-x_{t-1}\right)}^{2}+{\left(y_{t}-y_{t-1}\right)}^{2}}}+n_{1},\frac{\delta \left(t\right)\cdot d\cdot G\cdot \left(x_{t}-x_{t-1}\right)}{\sqrt{{\left(x_{t}-x_{t-1}\right)}^{2}+{\left(y_{t}-y_{t-1}\right)}^{2}}}+n_{2}\right)$$
8. **Data tampering**: The attacker replaces the state measurement value *P* of the target *M* uploaded by the radar station at time *t* with $P^{\prime }$, where
  (22) $$P^{\prime }=P+\Delta P$$
9. **Enter the next action cycle**: The attacker repeats the above process until the operation is completed.

### 4.2. Multi-Link Control Scenario

In scenarios involving coordinated attacks by multiple attackers, this can be viewed as a combination of individual link controls. When different or identical attack methods are employed on different links to achieve mutual coordination, the potential consequences could be significantly more severe.

Increasing the number of radar sites within a network is a classic mitigation measure. The addition of radar nodes signifies an overlay of information sources. After processing by fusion algorithms, this can more effectively mitigate the impact of noise on measurements. In such cases, attackers might be inclined to adopt coordinated attacks under multi-link control. Here, two potential risks are illustrated:

Degradation: Attackers might employ “null value” attacks on one or several links, effectively severing these links. This reduces the radar network, during fusion processing, to what is essentially a single-node or dual-node radar system, thereby lowering the difficulty of the attack.

Superposition: Attackers might apply the same attack method to multiple links. Through the appropriate coordination of attack intensity, they can reinforce the attack’s effect.

## 5. Simulation Experiment

### 5.1. Single-Link Control Scenario

#### 5.1.1. Experimental Background

To verify the impact of the different data tampering methods on the fused tracks of a radar network, a simulation experiment was designed as follows. It was assumed that the current radar network adopted a centralized networking architecture. For the convenience of analysis, the radar network was composed of two radar sites, A and B, and a fusion center. It was also assumed that the attacker had hijacked the communication link between radar site B and the fusion center through a man-in-the-middle attack. Then, a simulation of the results of different false data injection attacks was conducted.

**Figure 5:**
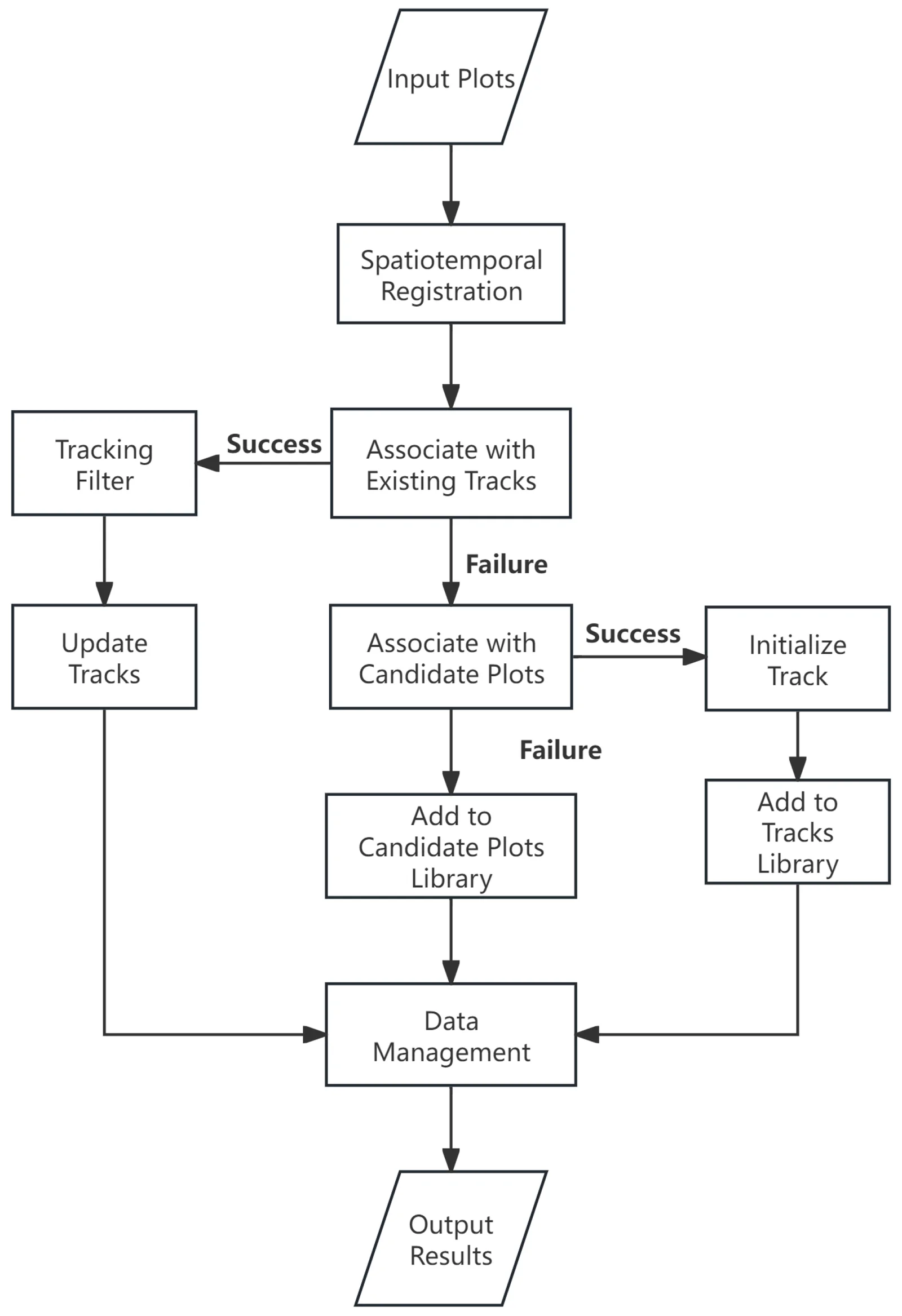
The plot processing flow at the fusion center.

The plot processing flow at the fusion center is illustrated in Figure 5 below:

In this setup, the three radars had the same system configuration and accuracy, with a data reporting period of 2 s. The tracking and filtering part of the fusion center employed parallel Kalman filtering. The attenuation coefficient of the optical fiber channel was 0.2 db/km and the transmission attenuation was 1 dB. In addition, to simulate packet loss caused by occasional bit error or equipment failure of the optical fiber link, the packet loss rate was assumed to be $1\times 10^{-5}$.

It was assumed that the target initially moves at a constant velocity in a straight-line path, starting from the coordinates (5000, 5000), with a velocity vector of (100, −100). In the later stage, it made a turn at a constant speed, resulting in an overall fish hook-shaped trajectory.

The green diamond-shaped plot points represented the true flight trajectory of the target. The orange “x”-shaped plot points marked the plot information uploaded by radar A. The black “x”-shaped plot points indicated the tampered plot information uploaded by radar B after modification. The circular plot points showed the fused tracks obtained at the central station. If there was a phenomenon of track splitting, different colors were used to distinguish between the different tracks. The computer configuration used for this experiment was as follows: The operating system was Windows 10 64-bit; the software platform was PyCharm 2023 with Python 3.9; and the computer used in the experiment was equipped with an Intel(R) Core(TM) i7-8565U CPU.

This experiment focused on the effectiveness of the attack; therefore, accumulated position offset (ACPO) and average position offset (AVPO) were selected as evaluation indicators.

This simulation experiment consisted of six experiments, the first five of which were no attack, null value attack, flip attack, scaling attack, and offset attack, while the last one used the false data construction algorithm proposed in this paper.

#### 5.1.2. Experimental Result

**Figure 6:**
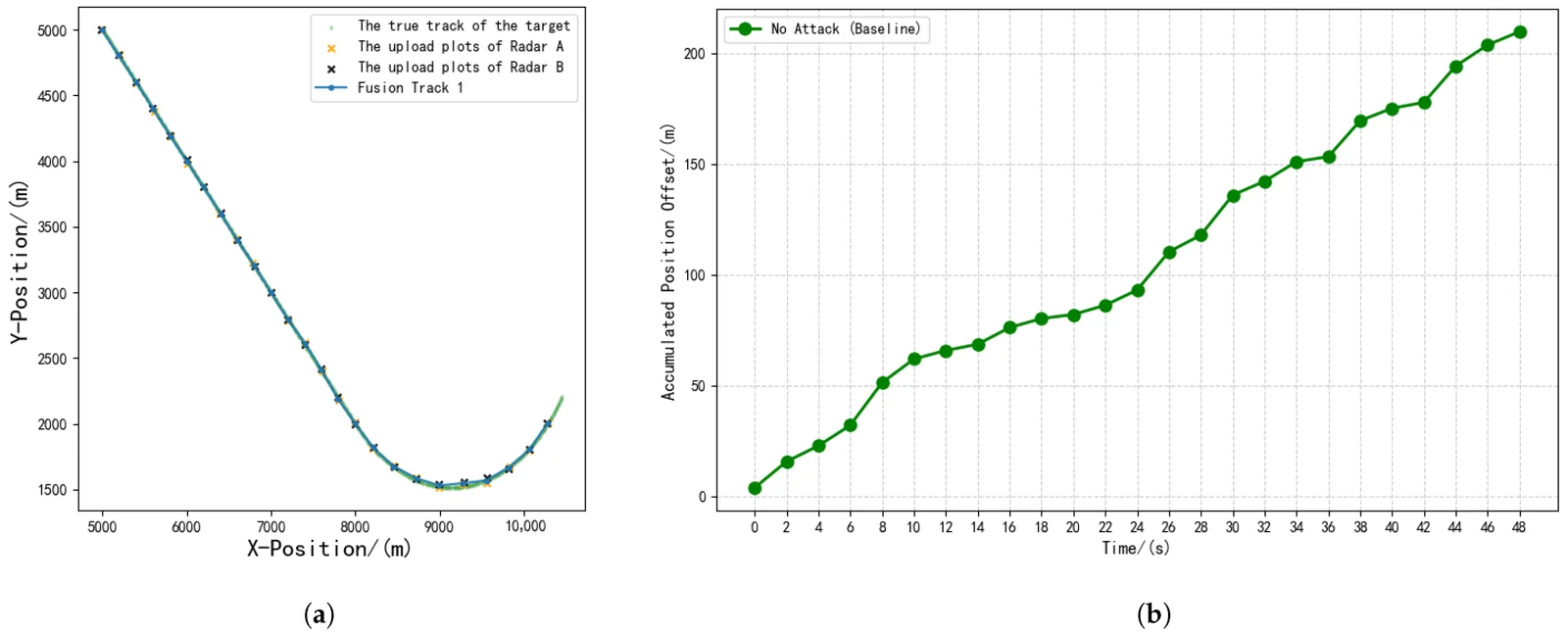
Experiment 1 results (no attack). (**a**) Comparison between fusion track and real track. (**b**) Accumulated position offset between fusion track and real track.

The results of Experiment 1 indicated that in the absence of attacks, the fused result generated by the fusion center aligned well with the actual track. The accumulated position offset within 50 s was merely 209.57 m, with an average position offset of only 8.38 m. The experimental results are shown in Figure 6.

**Figure 7:**
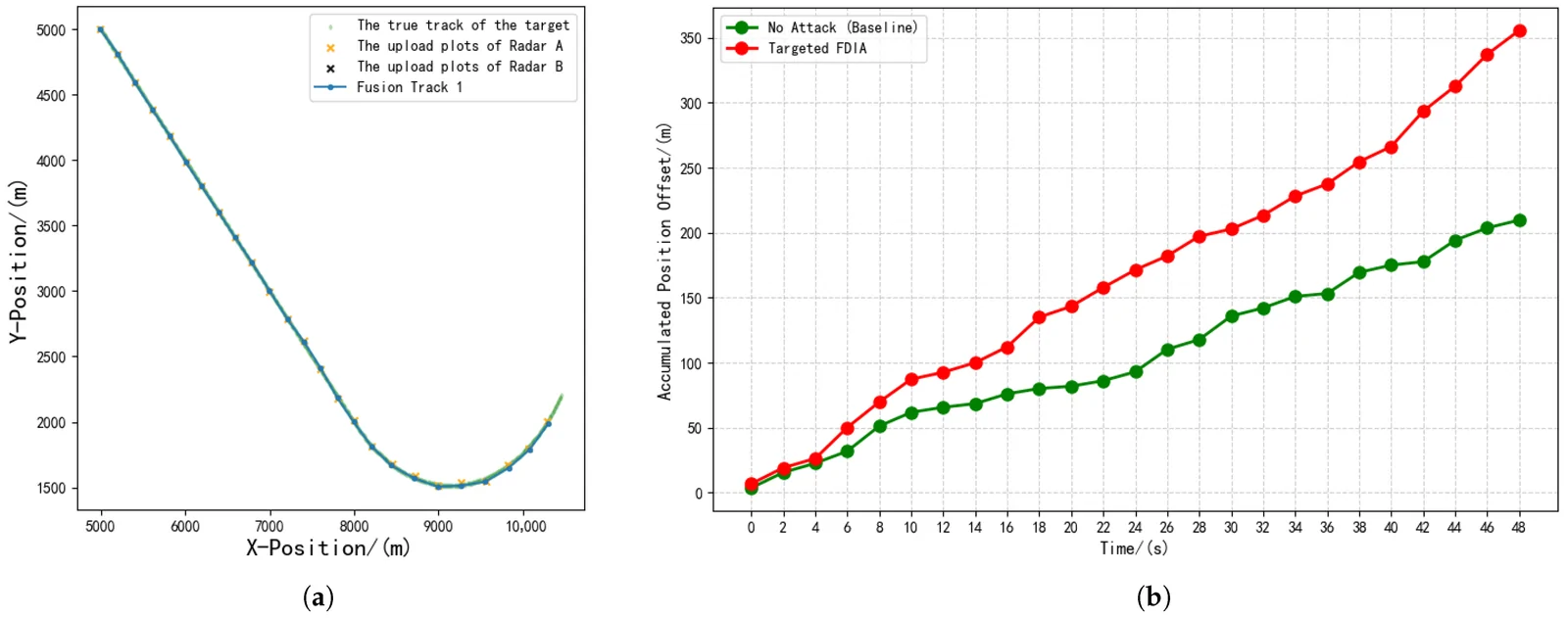
Experiment 2 results (null value attack). (**a**) Comparison between fusion track and real track. (**b**) Accumulated position offset between fusion track and real track.

In Experiment 2, a null attack was implemented, where the plot data uploaded from radar site B was blocked. The fusion center’s result then displayed only the orange x-shaped plots from radar site A. Despite having information from only one radar available, the final fused result still closely matched the actual track, with a cumulative offset of 355.47 m and an average single-step offset of 14.22 m. This demonstrated the robustness of the radar network. The experimental results are shown in Figure 7.

**Figure 8:**
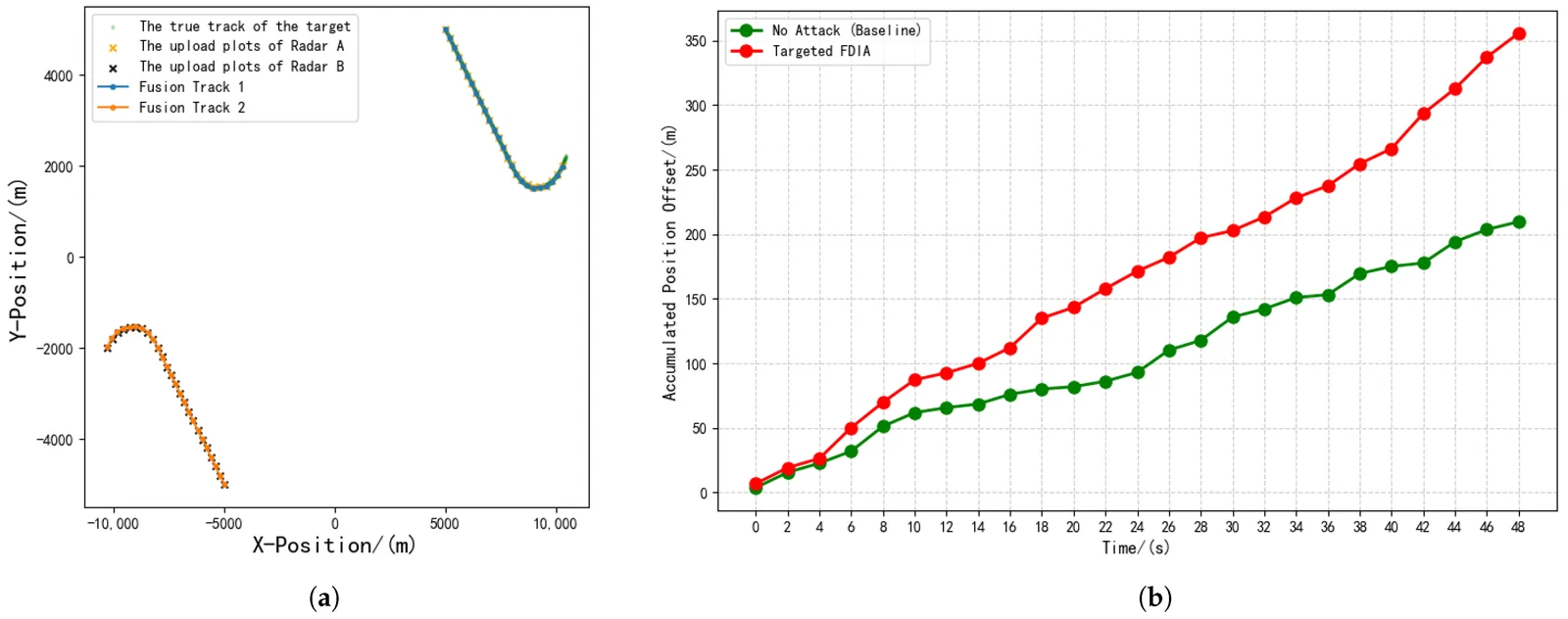
Experiment 3 results (flip attack). (**a**) Comparison between fusion track and real track. (**b**) Accumulated offset between fusion track and real track.

Experiment 3 involved a flipping attack, where the original plots from radar site B were flipped, resulting in a track that was symmetrical, with radar site B as the center. The simulation results showed a significant deviation between this track and the actual one, so it was treated as a completely new track at the fusion center. However, due to the presence of the orange x-shaped plots from radar site A, there was still a track that highly coincided with the target track. The final cumulative offset was 355.47 m and the average single-step offset was 14.22 m, the same as in Experiment 2. This indicated that this method could generate false tracks but had little effect on existing ones. The experimental results are shown in Figure 8.

In Experiment 4, a scaling attack was implemented. Specifically, the plot coordinates uploaded from radar site B were scaled down by a very small margin, approximately 1% of the coordinate values. As a result, the tampered plots remained near the original track and were associated with the same target’s plots at the fusion center. Consequently, the fused tracks after track filtering exhibited slight offsets. The final accumulated position offset was 1128.72 m and the average position offset was 45.15 m. The simulation results showed that the fused track at this point could no longer fully coincide with the actual track, but they were still quite similar. Although this attack achieved the effect of disrupting the original track, the impact was not strong enough.

Additionally, in this experiment, the scaling margin was increased to 5%, five times the original margin. The simulation results revealed that the tampered plots from radar site B deviated significantly from the actual track and were treated as a new track. If the scaling margin was further increased to 50%, the effect became even more pronounced. However, due to the presence of the orange x-shaped plots from radar site A, there was still a track that highly coincided with the target track. The final accumulated position offset remained at 355.47 m and the average position offset remained at 14.22 m.

**Figure 9:**
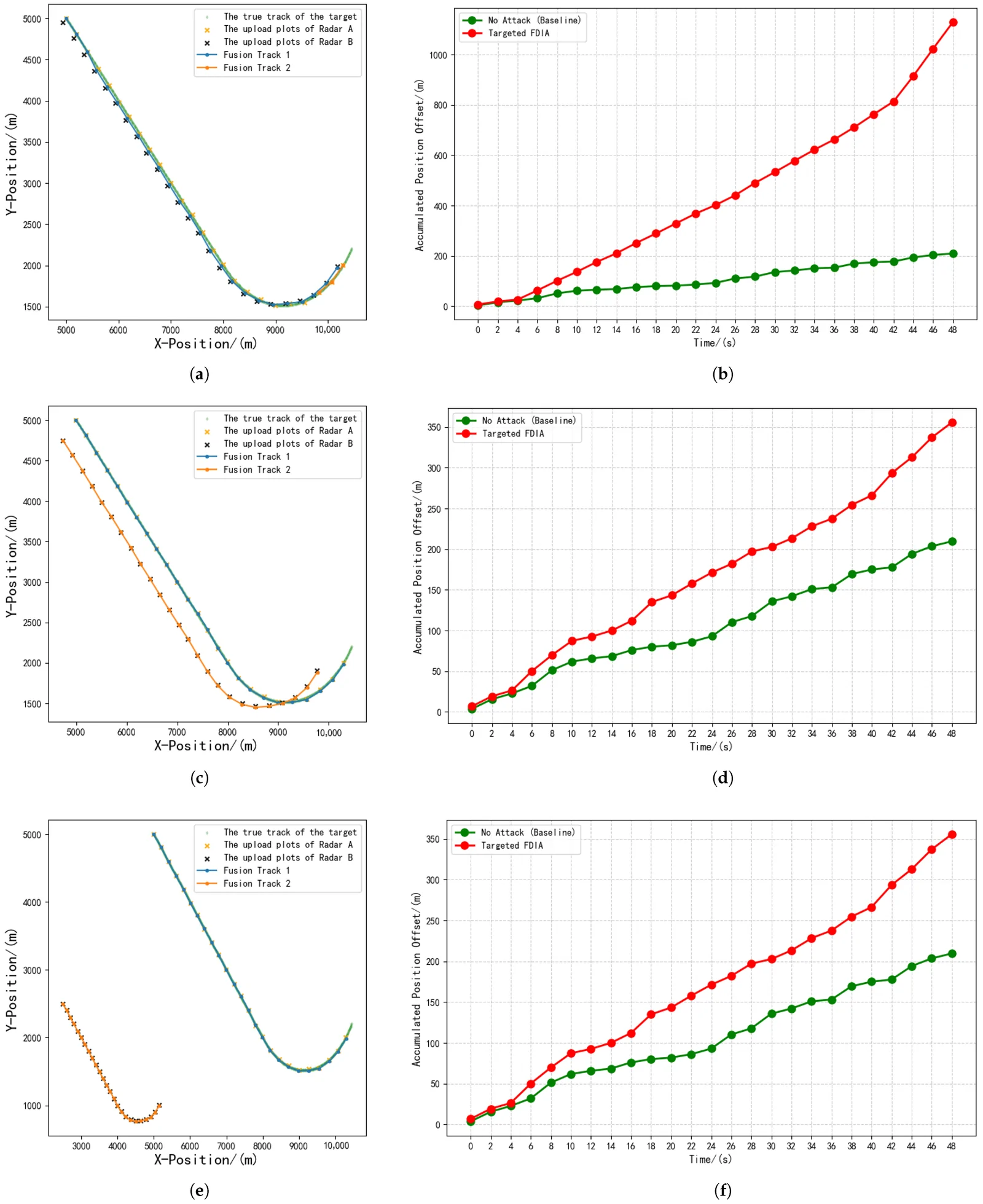
Experiment 4 results (scaling attack). (**a**) Comparison between fusion track and real track (shrinking by 1%). (**b**) Accumulated position offset between fusion track and real track (shrinking by 1%). (**c**) Comparison between fusion track and real track (shrinking by 5%). (**d**) Accumulated position offset between fusion track and real track (shrinking by 5%). (**e**) Comparison between fusion track and real track (shrinking by 50%). (**f**) Accumulated position offset between fusion track and real track (shrinking by 50%).

In summary, this method could disrupt the original track when the scaling margin was small, but the visual change in the track was not obvious. When the scaling margin was large, it could generate new false tracks and also affect the radar operator’s judgment of the target’s speed, but it could not influence the existing tracks. The experimental results are shown in Figure 9.

Experiment 5 involved an offset attack, with both the horizontal and vertical coordinate offsets set to 500 m. The simulation results showed two parallel tracks, one coinciding with the actual track and the other parallel to it. This attack also achieved the effect of generating false tracks.

**Figure 10:**
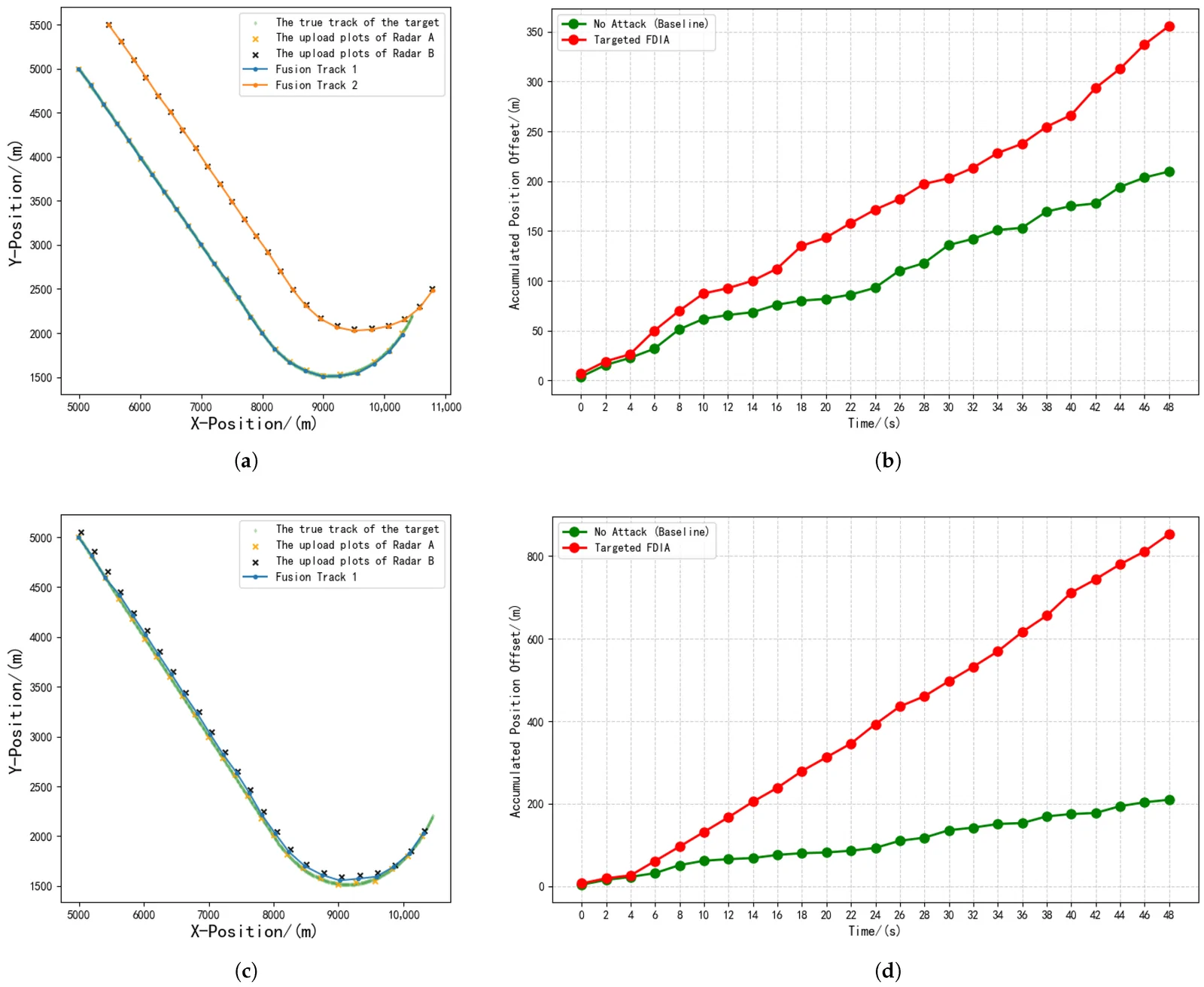
Experiment 5 results (offset attack). (**a**) Comparison between fusion track and real track (offset 500 m). (**b**) Accumulated position offset between fusion track and real track (offset 500 m). (**c**) Comparison between fusion track and real track (offset 50 m). (**d**) Accumulated position offset between fusion track and real track (offset 50 m).

If the translation offset was reduced to 50 m, the simulation results showed only one track. This was because the tampered plots remained near the original track and were associated with the same target’s plots at the fusion center. Consequently, the fused tracks after track filtering exhibited slight offsets. The simulation results indicated that the fused track at this point could no longer fully coincide with the actual track. The accumulated position offset was 852.84 m and the average position offset was 34.11 m, but they were still quite similar. Although this attack achieved the effect of disrupting the original track, the impact was not strong enough. The experimental results are shown in Figure 10.

In summary, this method could disrupt the original track when the offset was small, but the visual change in the track was still not obvious. When the offset was large, it could generate new parallel false tracks but still could not influence the existing tracks.

**Figure 11:**
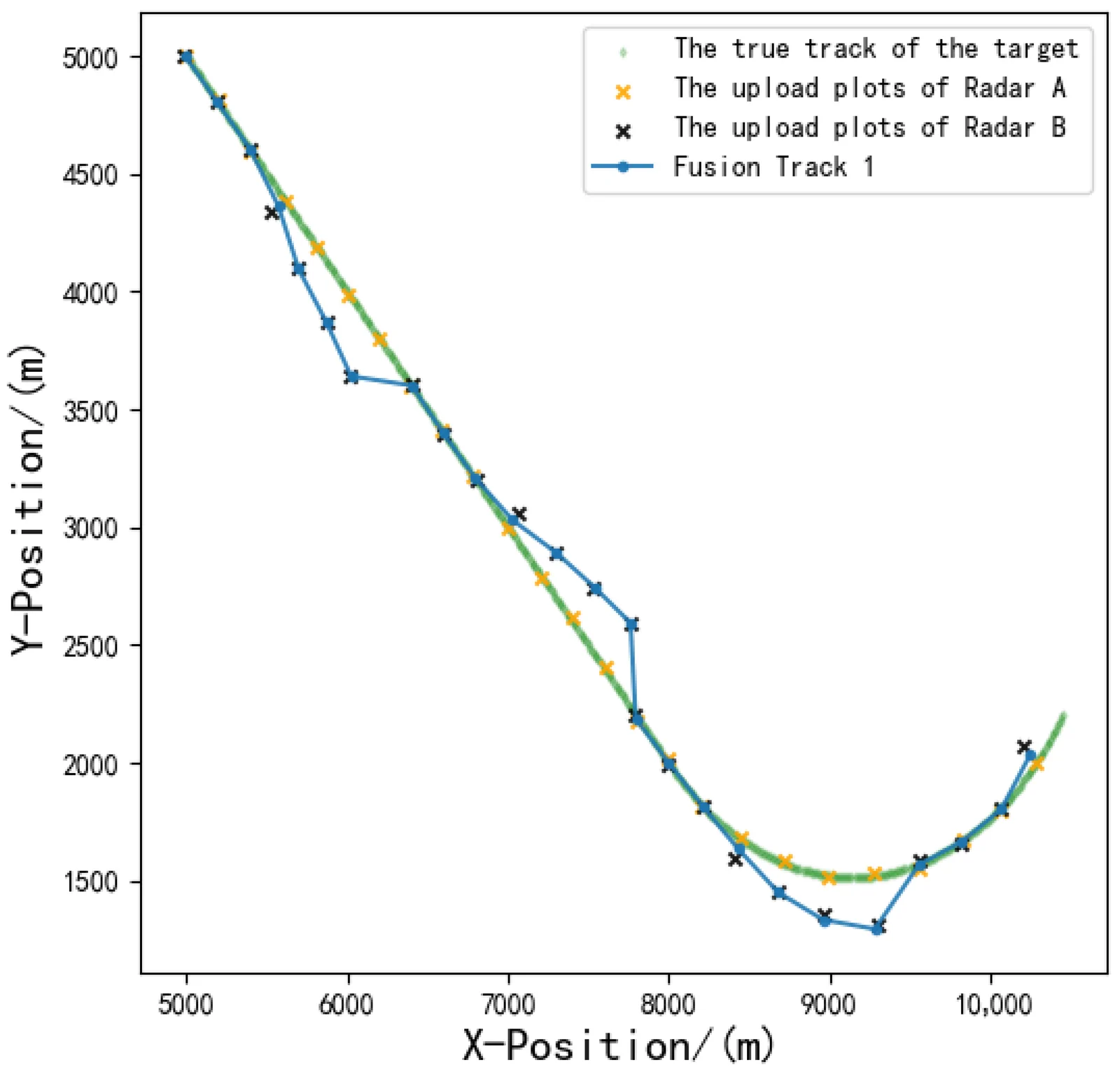
Comparison between fusion track and real track (method proposed in this paper).

**Figure 12:**
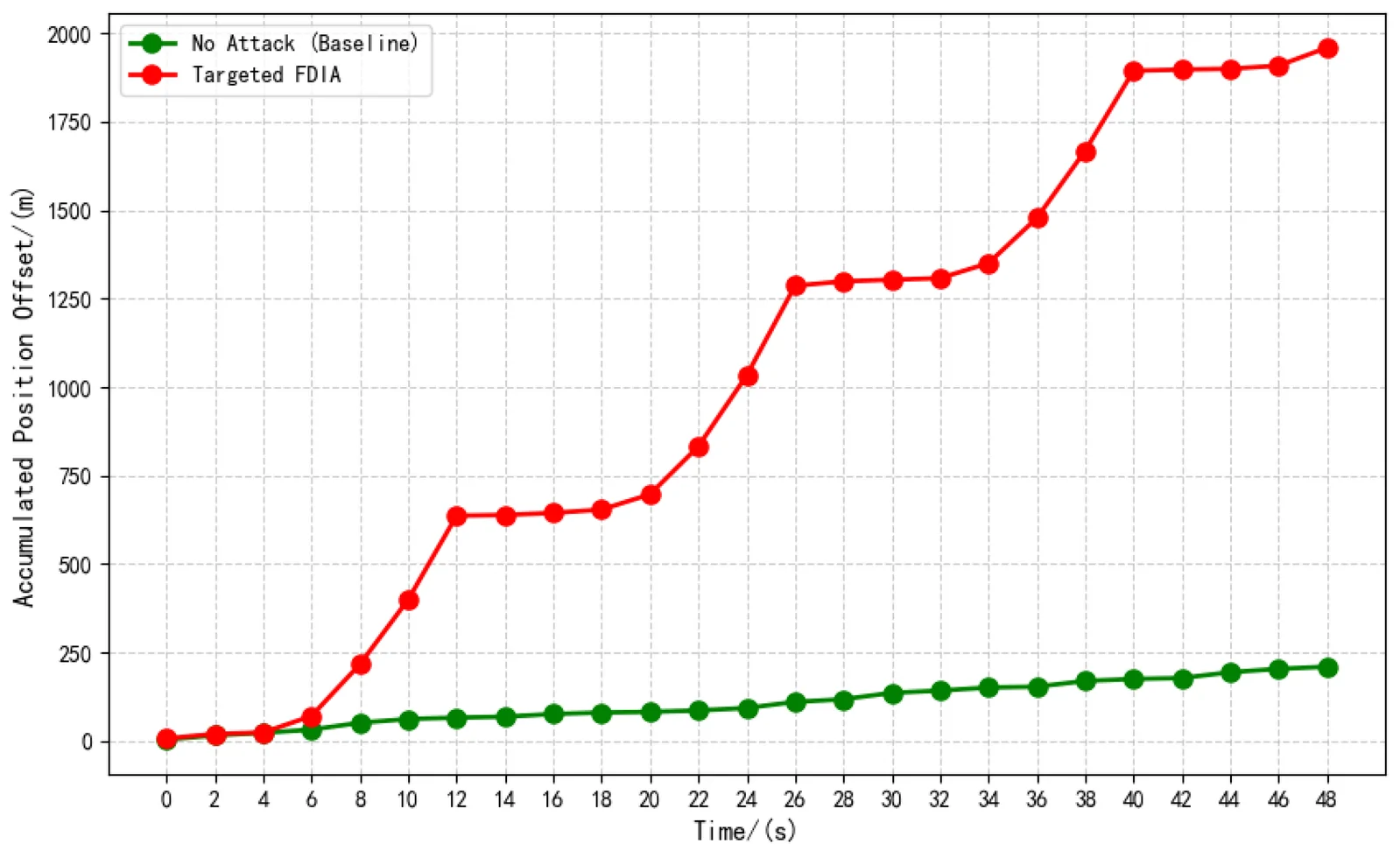
Accumulated position offset between fusion track and real track (method proposed in this paper).

Experiment 6 employed the data construction method proposed in this paper, which also involved tampering with the original data. This attack method adopted the ideas of periodically and intermittently injecting false data, linearly increasing the offset over time, and periodically flipping the offset direction. The simulation results showed that due to the periodic and intermittent injection of false data and the linearly increasing offset over time, the fusion center generated only one track. This track sometimes coincided with and sometimes deviated significantly from the actual track. Meanwhile, by intermittently changing the offset direction, the track could be distorted left and right, creating the illusion of continuous target maneuvering. At this point, the accumulated position offset reached 1958.8 m and the average position offset reached 78.35 m, with a significant visual change, effectively disrupting the original track. The experimental results are shown in Figure 11 and Figure 12. A summary of six experimental results is shown in Table 1.

### 5.2. Multi-Link Control Scenario

#### 5.2.1. Experimental Background

We refer to this supplementary experiment as Experiment 7. To better align the experiment with real-world scenarios, we increased the number of radar nodes as a mitigation measure. We also designed a multi-attacker scenario, increasing the number of attackers to investigate the impact of coordinated attacks. The scenario assumed a radar network composed of three radar sites (A, B, C) and one fusion center; other conditions remained the same as in the previous experiment. Additionally, Attacker 1 tampered with the data from radar site B while Attacker 2 tampered with the data from radar site C. Data from radar site A remained normal. The position coordinates of each radar station were (−5000, 0), (0, 5000), and (5000, 0) and the position coordinates of the fusion center were (0, 0). Each radar station was 5km away from the fusion center and optical fiber was used for communication between them.

First, the experiment was conducted under no-attack conditions to establish a reference baseline.

Coordinated Strategy 1: Attacker 1 employed the method proposed in Section 4 of this paper, with an initial coefficient α of 0.9 and a modulation slope *k* of 0.5. Attacker 2 employed a “null-value” attack.

Coordinated Strategy 2: Both Attacker 1 and Attacker 2 employed the method proposed in Section 4, with initial coefficients α of 0.9 and 1.8 and modulation slopes *k* of 0.5 and 0.6, respectively.

The green diamond-shaped plot points represented the true flight trajectory of the target. The orange “x”-shaped plot points marked the plot information uploaded by radar A. The black “x”-shaped plot points indicated the tampered plot information uploaded by radar B after modification. The red “x”-shaped plot points indicated the tampered plot information uploaded by radar C after modification.

#### 5.2.2. Experimental Results

**Figure 13:**
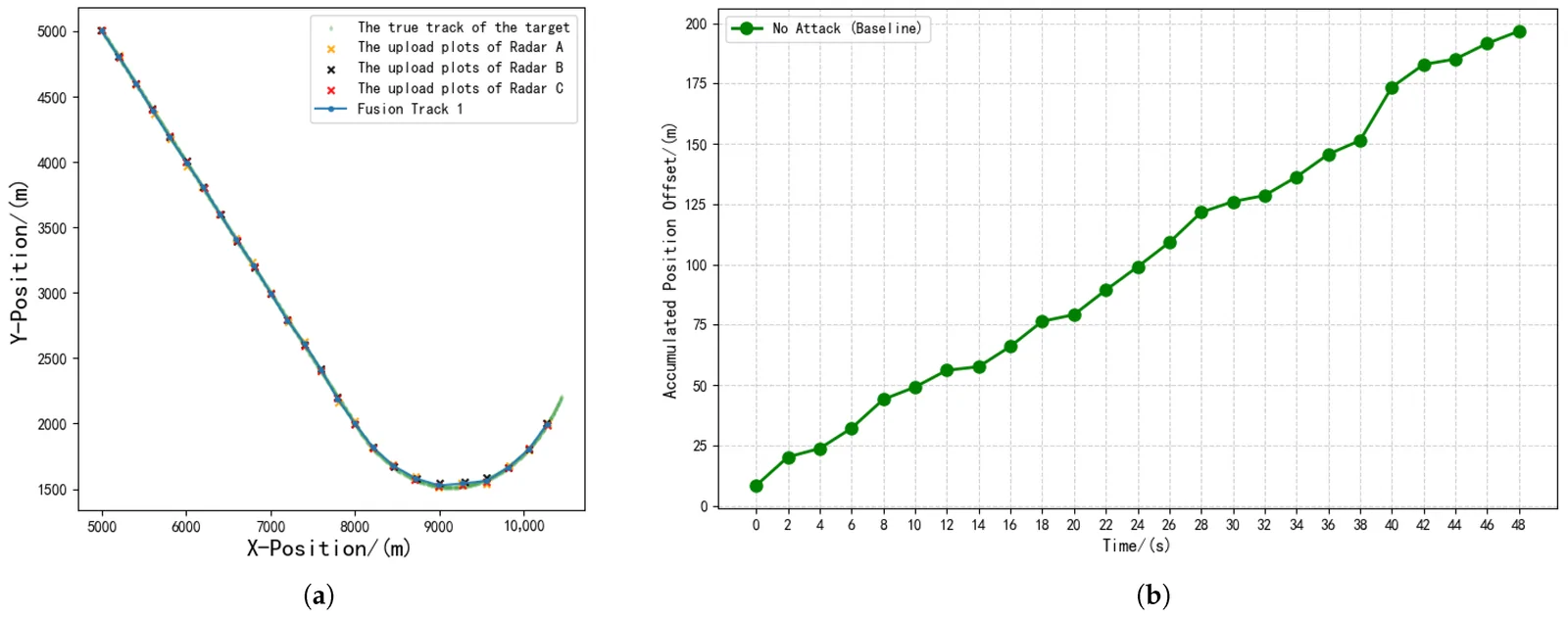

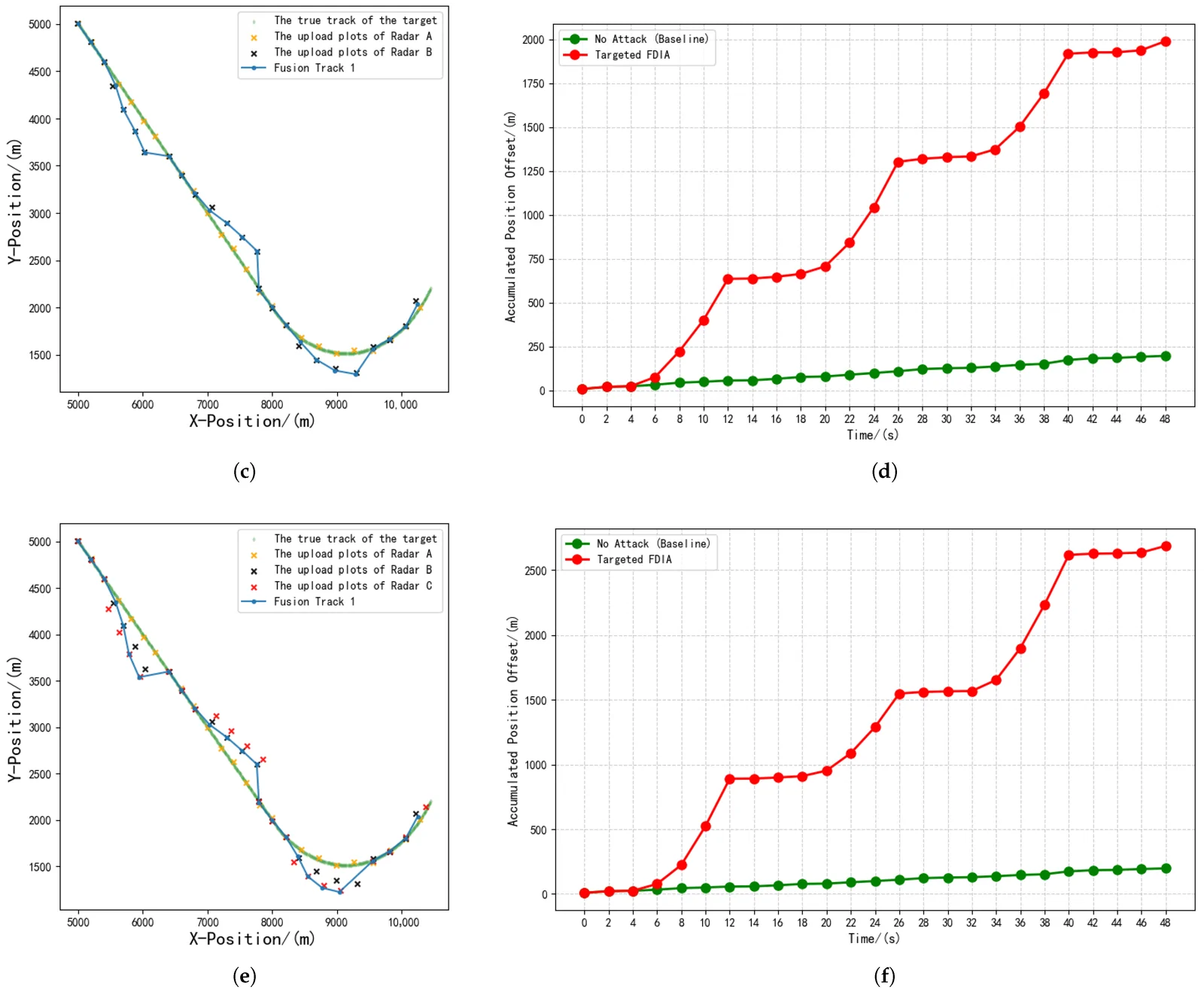
Experiment 7 results. (**a**) Comparison between fusion track and real track (no attack). (**b**) Accumulated position offset between fusion track and real track (no attack). (**c**) Comparison between fusion track and real track (Coordinated Strategy 1). (**d**) Accumulated position offset between fusion track and real track (Coordinated Strategy 1). (**e**) Comparison between fusion track and real track (Coordinated Strategy 2). (**f**) Accumulated position offset between fusion track and real track (Coordinated Strategy 2).

The results in Figure 13 and Table 2 show that under no-attack conditions, the fused result generated by the fusion center aligned well with the true target track. The cumulative offset over 50 s was only 196.75 m and the average step offset was only 7.87 m. Under Coordinated Strategy 1, the cumulative offset over 50 s was 1989.06 m, with an average step offset of 79.56 m. Under Coordinated Strategy 2, the cumulative offset over 50 s reached 2687.93 m, with an average step offset of 107.52 m.

## 6. Discussion

The results of Experiment 2 demonstrated that null attacks, which involved merely truncating the communication link between a single node in the radar network and the fusion center, did not affect the normal target tracking capability of the radar network. Due to the reduction in information sources, there could be a slight increase in the tracking error of the radar network, as evidenced by the increase in the average position offset from 8.38 m to 14.22 m. However, this is tolerable for real-world scenarios and does not disrupt existing tracks. This result also showcases the robustness of the radar network.

The results of Experiment 3 indicated that flip attacks could generate new false tracks but failed to disrupt existing tracks.

The results of Experiments 4 and 5 revealed that scaling/offset attacks could disrupt original tracks when changes were small, but the visual changes in the tracks were minor. Large changes could generate new false tracks but failed to disrupt the original ones. This was because small changes kept tampered points near the original track, allowing association with the same target. Consequently, the fused track after track filtering experienced a minor deviation. However, changes that exceeded the association gate prevented tampered points from associating with the original target. These points initialized as new tracks, creating false targets.

This finding differs from those of previous studies, which suggested that a larger attack offset leads to a larger deviation. However, this was based on the assumption that the false data could participate in the tracking filtering algorithm. In reality, due to the influence of track initialization and data association, within a certain threshold, a larger attack offset yields a larger deviation, but, beyond this threshold, the effect drops sharply.

The results of Experiment 6 showed that the false data construction method proposed in this paper could induce a significant deviation in the original track without splitting it, as evidenced by the increase in the average position offset from 8.38 m to 78.35 m. Additionally, it could cause the track to twist left and right, creating the illusion of continuous target maneuvering with noticeable visual changes. This attack method adopted the ideas of periodically and intermittently injecting false data, linearly increasing the offset over time, and periodically flipping the offset direction. Moreover, since this method only tampered with the original data, it did not cause fluctuations in traffic, demonstrating that constructing false data in a targeted manner can simultaneously achieve effectiveness and stealthiness.

The results of Experiment 7 indicated that adding radar nodes reduced AVPO by 6.09%, improving localization accuracy. Coordinated Strategy 1 mirrored the Experiment 6 results because Attacker 2’s “null-value” attack degraded the network to Experiment 6 conditions. Coordinated Strategy 2 enhanced the impact markedly, increasing AVPO by 37.23%. This substantial improvement stemmed from both attackers employing the same attack method while coordinating their attack intensities: Attacker 1 pulled the track off course and Attacker 2 amplified the deviation. Their closely coordinated attacks produced a compounding, reinforcing effect, revealing the significant risks posed by multi-attacker coordinated attacks.

In summary, the current radar network faces potential cybersecurity risks, including the possibility of attackers executing false data injection attacks (FDIAs) through man-in-the-middle attacks (MITMAs). If successful, attackers could easily manipulate data to create false tracks or alter existing ones. Moreover, by crafting targeted false data, they could significantly deviate original tracks without splitting them—all under single-link control and without increasing traffic. Since this leaves traffic volume unchanged, conventional detection methods based on traffic analysis would be largely ineffective, highlighting an urgent need for corresponding defense measures. In addition, if multiple attackers adopt a coordinated attack, it can cause more serious consequences, such as greater displacement.

## 7. Conclusions

This study analyzes and simulates the security risks of radar networks from a cyberspace security perspective. The findings indicate a significant security risk where attackers can perform false data injection into the fusion center of a radar network via man-in-the-middle attacks. This simulation results show that if attackers succeed in false data injection, even with only single-link control, they can create false tracks or cause slight deviations in existing tracks through simple data manipulation. Furthermore, by deliberately crafting false data, attackers can induce substantial deviations in original tracks without splitting them, making the tracks twist left and right to simulate serpentine maneuvers. If multiple malicious attackers use the above method to carry out cooperative attacks, this will cause more serious deviation. This would severely degrade the radar network’s tracking capability and poses a major security risk. Notably, these attacks do not alter network traffic, rendering traditional traffic-based detection methods ineffective and highlighting the vulnerability of radar networks to such threats. The study provides a reference for future research on countermeasures.

Future work will focus on developing defensive measures. Firstly, efforts will concentrate on novel attack detection methods. Research will investigate subtle anomalous patterns within track data introduced by attacks, such as deviations in spatiotemporal correlation and kinematic consistency. Machine learning or statistical inference-based real-time detection models will be developed to identify suspicious data within the information received by fusion centers.

Secondly, improvements to fusion algorithms will be pursued. This involves researching and enhancing the inherent robustness of existing fusion algorithms, with a specific focus on developing algorithms capable of effectively suppressing the influence of outliers or significantly erroneous measurements. Alternatively, the introduction of non-radar information sources, such as ADS-B, for heterogeneous information fusion will be considered. Leveraging the redundancy and complementarity of information will enable the cross-validation of radar tracks, thereby improving the ability to identify spoofing attacks.

Thirdly, encrypted communication will be a key area. Research will be conducted on lightweight cryptographic algorithms or digital signature schemes suitable for radar networks. This aims to ensure the authenticity of data sources and the integrity of data during transmission, fundamentally preventing man-in-the-middle attacks from tampering with or injecting data.

## Figures and Tables

**Table 1:** Comparison of experimental results. Accumulated position offset (ACPO) and average position offset (AVPO) were selected as indicators.

Experiment	Type	ACPO	AVPO
1	No attack	209.57	8.38
2	Null value attack	355.47	14.22
3	Flip attack	355.47	14.22
	Scaling attack (1%)	1128.72	45.15
4	Scaling attack (5%)	355.47	14.22
	Scaling attack (50%)	355.47	14.22
5	Offset attack (500 m)	355.47	14.22
	Offset attack (50 m)	852.84	34.11
6	The method proposed in this paper	1958.84	78.35

**Table 2:** Table of Experiment 7 results. Accumulated position offset (ACPO) and average position offset (AVPO) were selected as indicators.

Experiment	Type	ACPO	AVPO
1	No attack	196.75	7.87
2	Coordinated Strategy 1	1989.06	79.56
3	Coordinated Strategy 2	2687.93	107.52

## Data Availability

The original contributions presented in this study are included in the article. Further inquiries can be directed to the corresponding author.

## Abbreviations

The following abbreviations are used in this manuscript:

MITM	Man-in-the-Middle
FDIA	False Data Injection Attack
ATC	Air Traffic Control
CPS	Cyber–Physical System
